# Simulated shift work schedules disrupts circadian rhythms and behavior in mice

**DOI:** 10.1152/function.012.2026

**Published:** 2026-10-01

**Authors:** Jun-Yi Duan, Xiao-Tong Huang, Ai-Yu Duan, Li Ma, Wei Chen, Qun Luo

**Affiliations:** Naval Medical Center, PLA Naval Medical University, Shanghai, People’s Republic of China

**Keywords:** circadian rhythm, clock gene, gut microbiome, mood, shift work

## Abstract

Shift work has become a major cause of circadian rhythm disruption in modern society. This study investigated the systemic consequences of shift schedules by subjecting mice to different light-dark (LD) cycles: non-24-h cycles (rapidly rotating *groups A* and *B*) and 24-h cycles (*group C* and control group). We performed behavioral tests, serum hormone measurements, transcriptomic analysis of suprachiasmatic nucleus (SCN) genes, and gut microbiome analysis. The results showed that the mice exhibited significant body weight gain after altering LD cycle compared with the control group. In groups exposed to non-24-h cycles, the circadian expression rhythms of core clock genes (*Per*, *Cry*, and *Bmal1*) in the SCN were lost. Gene Ontology and Kyoto Encyclopedia of Genes and Genomes enrichment analyses revealed that genes in the SCN losing rhythmicity were significantly enriched in metabolic and regulatory pathways, disrupting endocrine and metabolic rhythms and ultimately compromising health. The circadian rhythms of key hormones like melatonin and dopamine were also lost. Concurrently, these central disruptions drove peripheral pathology, including gut microbiota dysbiosis (altered Firmicutes/Bacteroidota ratio, reduced Verrucomicrobia) and a profound loss of gut microbial diurnal oscillations. Behaviorally, this multisystem discord manifested as significantly increased anxiety-like and depression-like behaviors and fatigue (*P* < 0.05), whereas spatial memory remained intact, indicating selective affective vulnerability. SCN collapse, together with hormonal chaos, desynchronizes the gut microbiome, potentially promoting inflammation and metabolic dysfunction that further disrupts central regulation. These findings provide a physiological foundation for understanding the health consequences of circadian disruption and may help guide the future design of healthier shift work schedules.

**NEW & NOTEWORTHY** Systematic comparison of multiple simulated shift schedules in mice reveals that non-24-h light–dark cycles, but not fixed 24-h shifted cycles, abolish suprachiasmatic nucleus core clock gene (*Per*, *Cry*, *Bmal1*) rhythms, disrupt melatonin and dopamine, and desynchronize gut microbiota at the phylum level. These central and peripheral disruptions selectively exacerbate anxiety- and depression-like behaviors without impairing spatial memory, revealing a hierarchical disintegration of circadian organization. The findings provide a physiological framework for evidence-based shift scheduling.

## Graphical abstract

**Figure:**
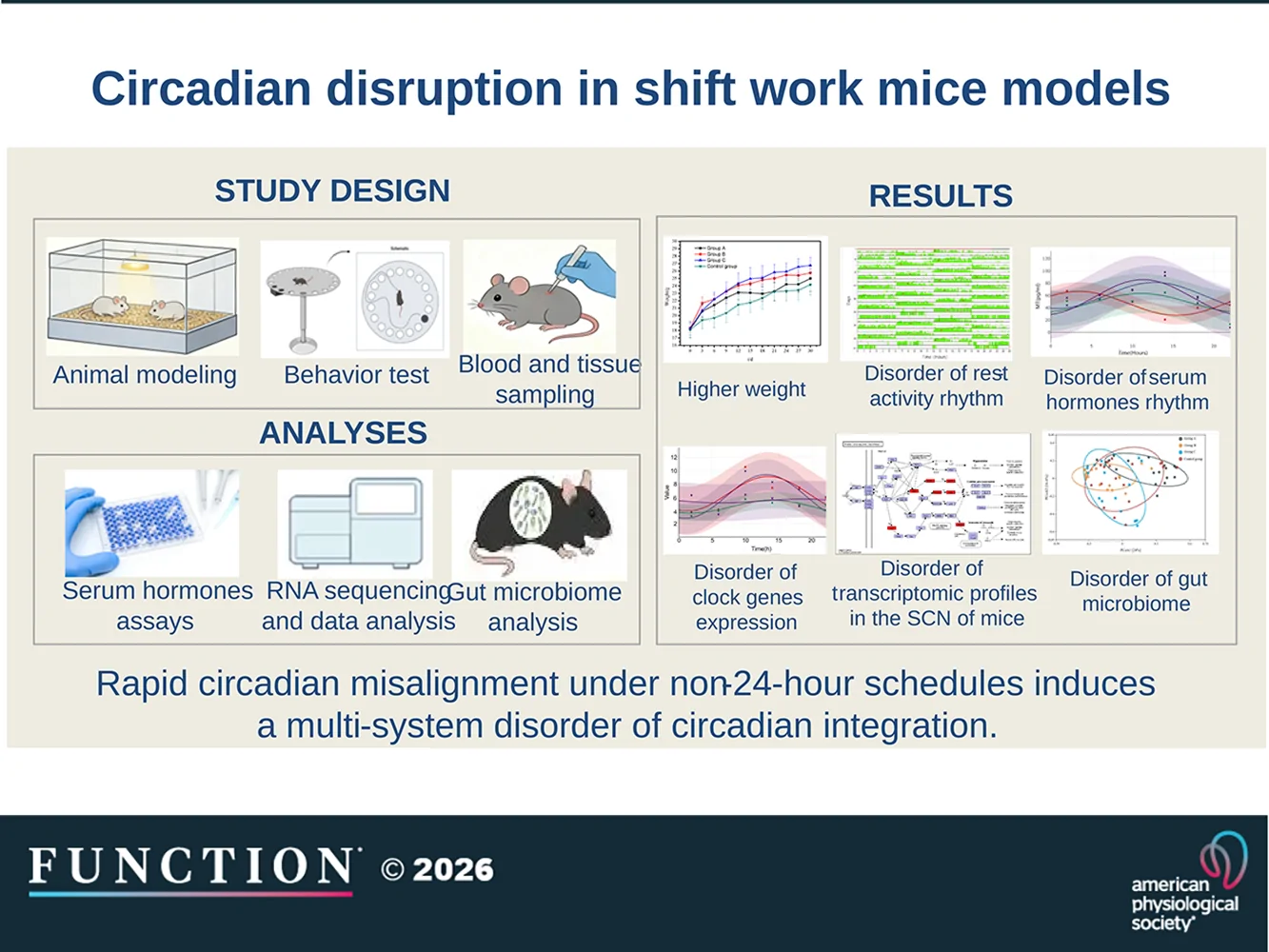

## INTRODUCTION

Circadian rhythms, which are primarily regulated by the suprachiasmatic nucleus (SCN) of the hypothalamus, orchestrate a broad spectrum of physiological and behavioral functions, including sleep-wake cycles, hormone secretion, metabolic activity, and core body temperature regulation (1). Human circadian clocks synchronize these daily physiological and behavioral rhythms with the 24-h rotation period of the Earth. Exposure to non-24-h cycles, which fall outside the entrainment range of the circadian system, can disrupt these rhythms, leading to misalignment and related disorders. In modern society, shift work has become a main cause of circadian rhythm disorders. Medical workers, military personnel, transportation operators, manufacturing workers, and other professions, due to the particularity and importance of their occupations and job requirements, have long been exposed to shift work, leading to circadian rhythm disorders and sleep problems. In some special environments, such as submarines and commercial ships, non-24-h watch schedules are often used (2). This necessity for persistent vigilance and rapid response relies heavily on personnel adhering to non-24-h schedules, which often involve rapid rotations, night shifts, and extended periods of alertness. Personnel working under such schedules experience misalignment between their work-sleep cycles and internal circadian timing, which would elevate health risks and adversely affecting mood, cognition, and performance (3). The health consequences for shift workers can be severe and systemic. Chronic circadian misalignment disrupts the body’s master clock, leading to a cascade of pathophysiological consequences (4). Coherent hormonal signaling collapses, and systemic inflammation rises. This internal desynchronization directly impairs cognitive function-manifesting as degraded alertness, slowed reaction times, and impaired decision-making, which critically compromises operational safety and mission effectiveness (5). Furthermore, this physiological strain reprograms the central nervous system toward disease-susceptible states, significantly increasing the risks of mood disorders like anxiety and depression, metabolic dysfunction, and gastrointestinal issues (6).

Epidemiological and interventional studies consistently link shift schedule characteristics to the degree of circadian misalignment and the severity of downstream health detriments, including metabolic dysfunction, cognitive impairment, and affective disorders (7). The systematic investigation of various shift schedules (e.g., rapid vs. slow rotation, 24-h cycle vs. non-24-h cycle) is fundamental for developing evidence-based strategies to protect shift worker health. Optimization of shift work schedules, grounded in circadian biology principles and targeted at the underlying pathology, is pivotal for safeguarding long-term health, preserving cognitive acuity, and sustaining workforce operational capacity, thereby establishing a proactive scientific foundation for evidence-based policy and practice.

This study altered light-dark cycles in mice to simulate different shift work schedules and investigated the effects of these schedules on activity levels, endogenous circadian rhythms, fatigue, cognition, psychological state, as well as immune and metabolic functions through behavior test, serum hormone assays, transcriptome analysis of SCN genes, and gut microbiome analysis.

## MATERIALS AND METHODS

### Animals

**Figure 1.**
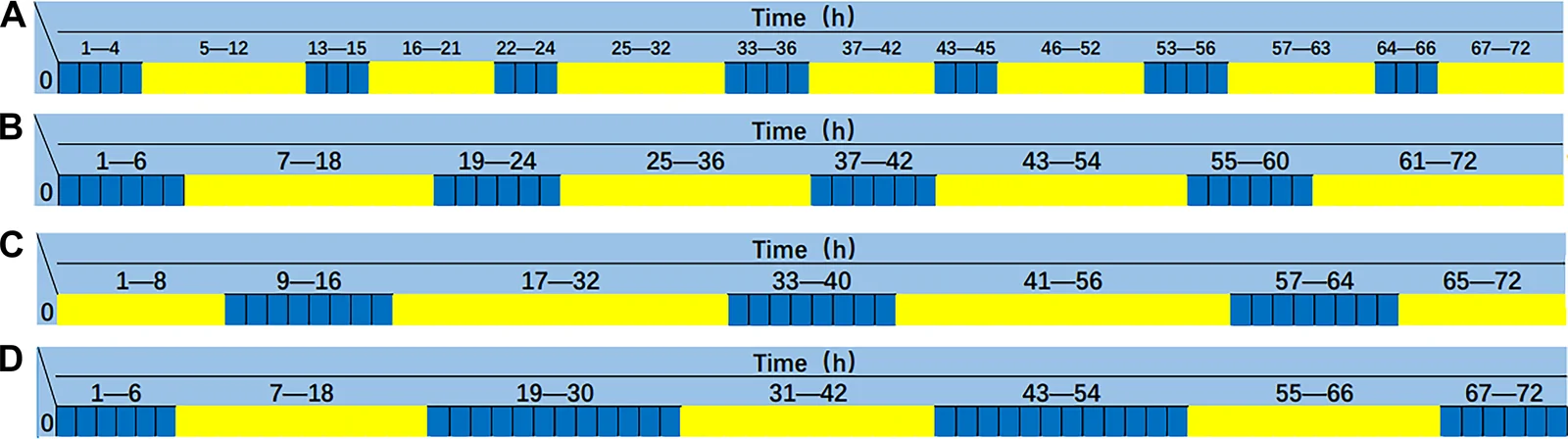
Light/dark (L/D) cycles for the four groups (each light-dark cycle spanned 72 h). The experiment lasted for 30 days, comprising a total of 10 complete cycles. *Group A*: 3–4 h D/6–8 h L (*A*), *group B*: 6 h D/12 h L (*B*), *group C*: 8 h D/16 h L (*C*), and control *group*: 12 h D/12 h L (*D*). Yellow: light time and blue: dark time.

A total of 144 male C57BL/6J mice were used in this study. Mice were housed in standard group cages at an ambient temperature of 23 ± 2°C with ad libitum access to food and water. All mice were acclimated to a natural 12-h light/12-h dark (LD) cycle for 10 days before the experiment. Subsequently, they were randomly divided into four groups (*n* = 36 per group): a control group maintained under standard 12-h light/12-h dark cycles (control group), and three experimental groups subjected to altered LD cycles: 3–4 h dark/6–8 h light (*group A*), 6 h dark/12 h light (*group B*), and 8 h dark/16 h light (*group C*). The non-24-h light-dark cycles used in this study were directly modeled on real-world rapidly rotating shift schedules used in military and maritime operations. *Group B* (6 h light/12 h dark) mimics the 6-h-on/12-h-off submarine watch schedule, which creates an 18-h day and induces circadian desynchronization. *Group A* (3–4 h light/6–8 h dark) represents a more fragmented variant used in high-intensity operations. *Group C* (8 h light/16 h dark) corresponds to a standard 24-h shift pattern. Their body weights were recorded at the beginning of the experiment and subsequently at 3-day intervals throughout the experiment period. To simulate long-term shift work, mice were subjected to LD cycle for a total of 30 days (Fig. 1). All animals were transferred to constant darkness (DD) at the end of the final LD cycle (*time 0 h*). The LD cycle regimen began when the mice were 9 wk old across groups. All mice were individually housed in home cages, and their activity was monitored using the ClockLab System (Actimetrics). All animal care and experimental procedures were conducted in accordance with the *Guide for the Care and Use of Laboratory Animals* standards adopted by the National Institutes of Health and approved by the Research Ethics Committee of PLA Naval Medical Center.

### Behavior Test

All behavioral tests were conducted in the same quiet laboratory on *day 31* of the experiment at the end of the final LD cycle. All animals from all four groups were released into constant darkness (DD). The tests were performed during the first 4 h after transfer to DD. For each behavioral test, six mice were randomly selected from each group (different mice were used for different tests), and the testing time was the same across all groups. The order of tests was as follows: Barnes maze test, Y-maze test, open-field test, tail suspension test, and treadmill test. The experimental setups (SanBio Co., Ltd., PR China) combined a camera with a video tracking system (Smart Co., Ltd.) for recording and analyzing animal behavior.

#### Barnes maze test.

The Barnes maze test (BMT) was used to assess spatial memory. The maze apparatus consists of a circular platform with 20 holes along its perimeter, 19 of which are empty and one containing a food reward. The camera above the platform detects mouse position, and the tracking system records behavioral trajectories and time spent in the maze. Mice were first trained once a day for 1 wk to locate the baited hole. During testing, each mouse was restricted to a central box on the Barnes maze for 20 s before being released to explore freely for 180 s. Recorded parameters included latency to first entry into the target hole and time spent in the target hole.

#### Y-maze test.

The Y-maze test (YMT) was used to evaluate short-term memory. The Y-shaped maze had three arms (each 350 mm long, 250 mm high, and 50 mm wide) arranged at 120° intervals. Mice were placed at the end of one arm and allowed to explore freely for 10 min. The sequence and total number of arm entries were recorded. A correct alternation was defined as three consecutive entries into three different arms. The number of correct alternations was recorded, and the spontaneous alternation ratio was calculated.

#### Open field test.

The open field test (OFT) was conducted to assess locomotor activity and anxiety-like behavior. The open-field apparatus consisted of an enclosed square arena (500 × 500 mm) with 300-mm-high walls. Mice were placed in the center of the arena and allowed to explore freely for 5 min. Anxiety-like behavior was assessed by the time spent in the center of the arena. Locomotor activity was evaluated by the total distance traveled.

#### Tail suspension test.

The tail suspension test (TST) was used to evaluate depression-like behavior. The test was conducted in a black acrylic box (600 × 430 × 550 mm). Each mouse was suspended by a hook attached 1 cm from the tip of its tail, hanging upside down 400 mm above the floor for 6 min. Immobility duration and high-activity duration were recorded using the video-tracking system.

##### Treadmill test.

The treadmill test (TT) was conducted to assess endurance and fatigue levels. Eight mice per group were trained to run on a treadmill at 0° inclination before testing. During the test, the treadmill inclination was set to 10° with an initial speed of 10 m/min for 30 min. The speed was then increased by 2 m/min every 15 min. The test continued until all mice were exhausted, and the total running distance for each mouse was recorded.

### Blood and Tissue Sampling

On the day after the final LD cycle, six mice per group were randomly sampled at six time points (0200, 0600, 1000, 1400, 1800, and 2200) over a 24-h period. Under isoflurane anesthesia, eyeball blood was collected into ice-cold 1.5 mL centrifuge tubes. Blood samples were centrifuged (6,000 rpm, 4°C, 10 min) to separate serum, which was stored at −80°C until analysis. For brain tissue collection, mice were euthanized by cervical dislocation. Brains were rapidly dissected and flash-frozen on dry ice. The SCN was bilaterally punched out from frozen brain tissue.

### Serum Hormones Assays

To assess circadian rhythms across groups, serum levels of melatonin (MT), cortisol (CT), dopamine (DA), and tumor necrosis factor alpha (TNF-α) were measured at the six time points using ELISA kits (Shanghai Bio-Light Company, PR China; MT: Cat. No. F11950; CT: Cat. No. F12206; DA: Cat. No. F12122; and TNF-α: Cat. No. F12044), according to the manufacturer’s protocol. Briefly, 10 µL of test sample mixed with 40 µL sample diluent was added to wells of a 96-well plate. Then, 100 µL of horseradish peroxidase-conjugate reagent was added to each well. The plate was covered and incubated for 60 min at 37°C. Wells were aspirated and washed five times with 400 µL wash solution. Subsequently, 50 µL each of chromogen *solutions A* and *B* were added, gently mixed, and incubated in the dark for 15 min at 37°C. The reaction was stopped by adding 50 µL stop solution per well. Optical density (OD) was measured at 450 nm within 15 min using a microplate reader.

### RNA Extraction, Library Construction, Sequencing, and Data Analysis

Total RNA of SCN was extracted using a commercial kit according to the manufacturer’s instructions (Guangdong Meg Gene Biotechnology Co., Ltd., Guangzhou, PR China; Cat. No. A005S). RNA degradation and contamination were assessed by 1% agarose gel electrophoresis. RNA concentration was measured using Qubit 4.0 (Thermo Fisher Scientific, Waltham) and NanoDrop One (Thermo Fisher Scientific, MA). RNA integrity was determined with the Agilent 4200 system (Agilent Technologies, Waldbronn, Germany). Libraries were constructed using the ALFA-SEQ RNA Library Prep Kit following the manufacturer’s instructions. Sequencing was performed on an Illumina platform, generating 150-bp paired-end reads.

Quality control was performed on the raw FASTQ data using Fastp (v0.23.2) to obtain clean reads. Ribosomal RNA sequences were then removed by aligning the clean data to the NCBI rRNA database with Bowtie2 (v2.4.5). The remaining reads were aligned to the reference genome using Hisat2 (v2.2.1), following download of the genome and annotation files from the respective database. Gene expression was quantified with RSEM (v1.3.3) and normalized using transcripts per million to enable cross-sample comparison. Finally, differential expression analysis between conditions was conducted with DESeq2 (v1.34.0), applying thresholds of false discovery rate (FDR) ≤ 0.05 and |log_2_(fold change)| ≥ 1 to identify significantly differentially expressed genes for downstream analysis (8). The Gene Ontology (GO; http://www.geneontology.org) database was used to identify and annotate significantly enriched biological processes. Kyoto Encyclopedia of Genes and Genomes (KEGG; http://www.genome.jp/kegg/) enrichment analysis was performed to identify pathways associated with disease progression. GO and KEGG enrichment analyses of differentially expressed genes were conducted using clusterProfiler (v4.2.2). GO terms and KEGG pathways with FDR ≤ 0.05 were considered significantly enriched.

### Gut Microbiome Analysis

On the day after the final LD cycle, fecal samples were collected at six time points (0200, 0600, 1000, 1400, 1800, and 2200) over 24 h and stored in 1.5 mL microcentrifuge tubes at −80°C. DNA was extracted using a DNA isolation kit (Guangdong Magigene Co., PR China; Cat. No. A009S). The bacterial 16S rRNA gene V4 region was amplified with primers 515 F and 806 R using the PCR conditions described in the document (30 cycles, annealing 52°C, amplicon size 290–310 bp). The amplicon library was prepared with the ALFA-SEQ DNA Library Prep Kit and sequenced on an Illumina or MGI platform (PE250; Guangdong Magigene Biotechnology Co., Ltd., Guangzhou, PR China). Raw paired-end reads were quality-filtered using fastp (v0.14.1) with sliding window quality trim (-W 4 -M 20). Primers were removed using cutadapt based on the 5′ and 3′ primer sequences. Clean reads were merged using usearch-fastq_mergepairs (v10) with a minimum overlap 16 bp and maximum mismatch 5 bp, followed by further quality filtering with fastp to obtain clean tags. Amplicon sequence variants (ASVs) were inferred using the DADA2 pipeline, which includes error correction, denoising, and chimera removal (9). Alternatively, operational taxonomic unit (OTU) clustering was performed at 97% similarity using UPARSE with chimera removal by uchime_ref. Representative sequences were taxonomically annotated using the SILVA (16S) reference database (default confidence threshold 0.8) via usearch-sintax or QIIME2’s feature-classifier. Sequences annotated as chloroplasts or mitochondria were removed from downstream analyses. To account for differences in sequencing depth, the ASV/OTU abundance table was rarefied to the minimum library size using the rrarefy function in R package vegan. Rarefaction curves were generated to confirm adequate sampling depth.

All statistical analyses were performed using R software (v5.1.3). Alpha diversity was calculated using usearch-alpha_div (version V10) and R package; group comparisons were performed using Kruskal–Wallis test (multiple groups) followed by Wilcoxon rank-sum test for pairwise comparisons, with FDR correction. Beta diversity was assessed using Bray–Curtis and Jaccard distance matrices, visualized by principal coordinate analysis (PCoA) using the R package vegan. Differences in community composition were tested by PERMANOVA (adonis, 999 permutations). When PERMANOVA was significant (*P* < 0.05), pairwise PERMANOVA with Benjamini–Hochberg false discovery rate (FDR) correction was conducted for post hoc comparisons.

### Statistical Analyses

GraphPad Prism 10 and SPSS 26 were used for statistical analysis. Data are presented as means ± SE. A *P* < 0.05 was considered statistically significant, and 0.05 < *P* < 0.1 was considered a trend. Circadian activity charts were plotted using ClockLab, and activity rhythm period and amplitude were analyzed using the Periodogram and fast Fourier transform methods.

The 24-h rhythmic data were analyzed using a classic cosinor model by Cosinor. bioRxivto calculate period, amplitude, and phase (10):

Y = M+A×cos(2π×(t – φ)/T)

where *M* is the mesor, *A* is amplitude, φ is acrophase (in hours), and *T* is the period.

## RESULTS

### Effects of Shift Schedules on Body Weight in Mice

**Figure 2.**
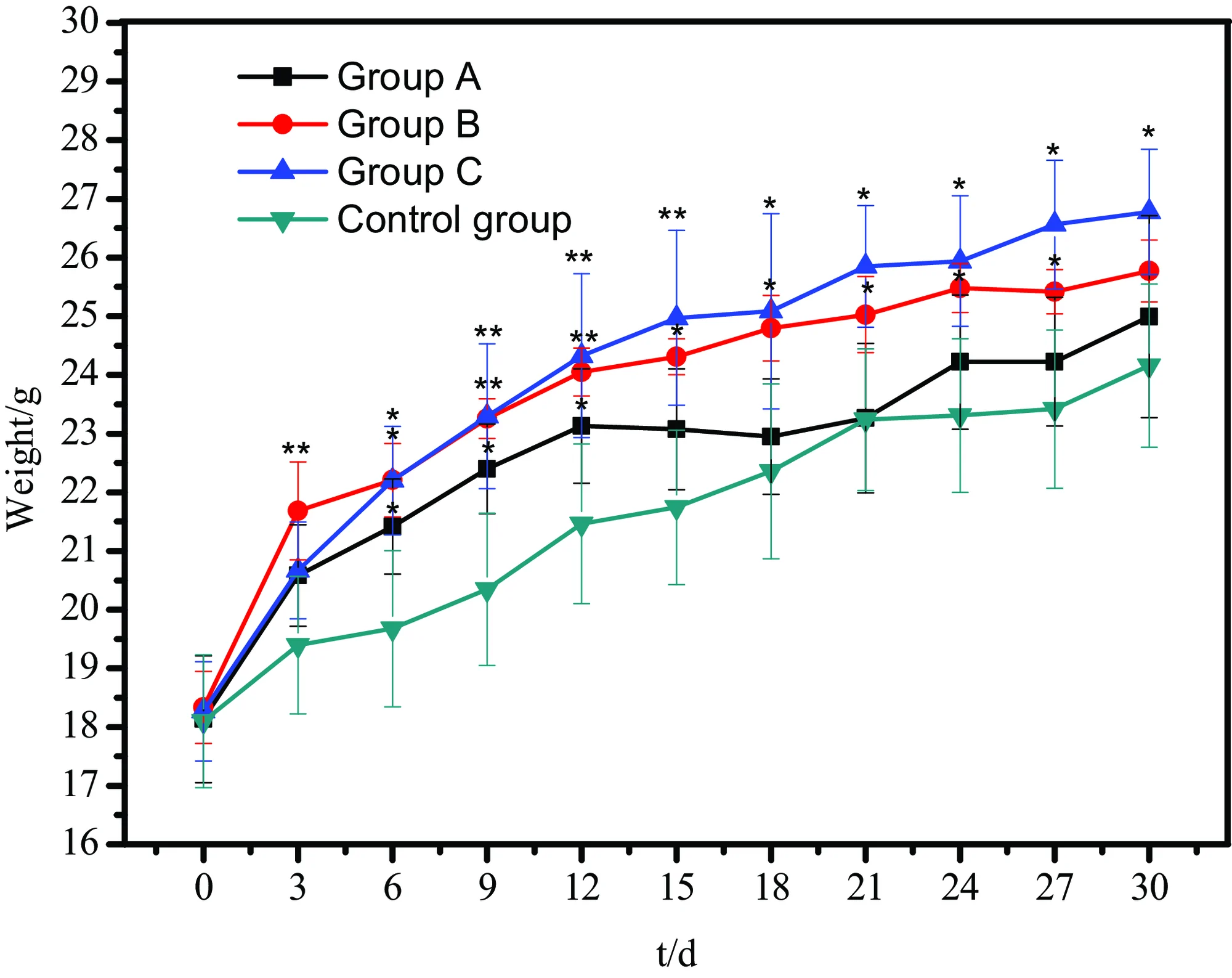
The body weight of mice in the four groups during the experimental period (data are presented as means ± SE. *P < 0.05, **P < 0.01 vs. control group; one-way ANOVA followed by Tukey’s post hoc test. *n* = 36 per group).

Changes in body weight of each group of mice are shown in Fig. 2. At the beginning of the experiment, no significant differences in body weight were observed among the four groups. During the experiment, the body weight of the mice in all groups increased gradually, and *groups B* and *C* were significantly higher than the control group (*P* < 0.05). For the first 12 days, the mice body weight in *group A* was also significantly higher than that in the control group (*P* < 0.05); however, after *day 12th*, the mice body weight was not as high as those in *group B* and *group C*, and had not statistically significant compared with the control group (*P* > 0.05). From *day 15* to *day 27*, the mice body weight in *group A* was significantly lower compared with *group B* and *group C*, respectively (*P* < 0.05), but no significant difference was found between *groups B* and *C* (*P* > 0.05). Body weight is a complex indicator of body health. Appropriate body weight is important to health. These data indicate that all experimental LD schedules led to increased body weight compared with the control group, but the effect was transient in *group A* (the most disrupted schedule), where weight gain plateaued after *day 12* and fell below that of *groups B* and *C*. The attenuation of weight gain in *group A* may reflect a differential metabolic response to more severe circadian disruption.

### Effects of Shift Schedules on Rest-Activity Rhythm in Mice

**Figure 3.**
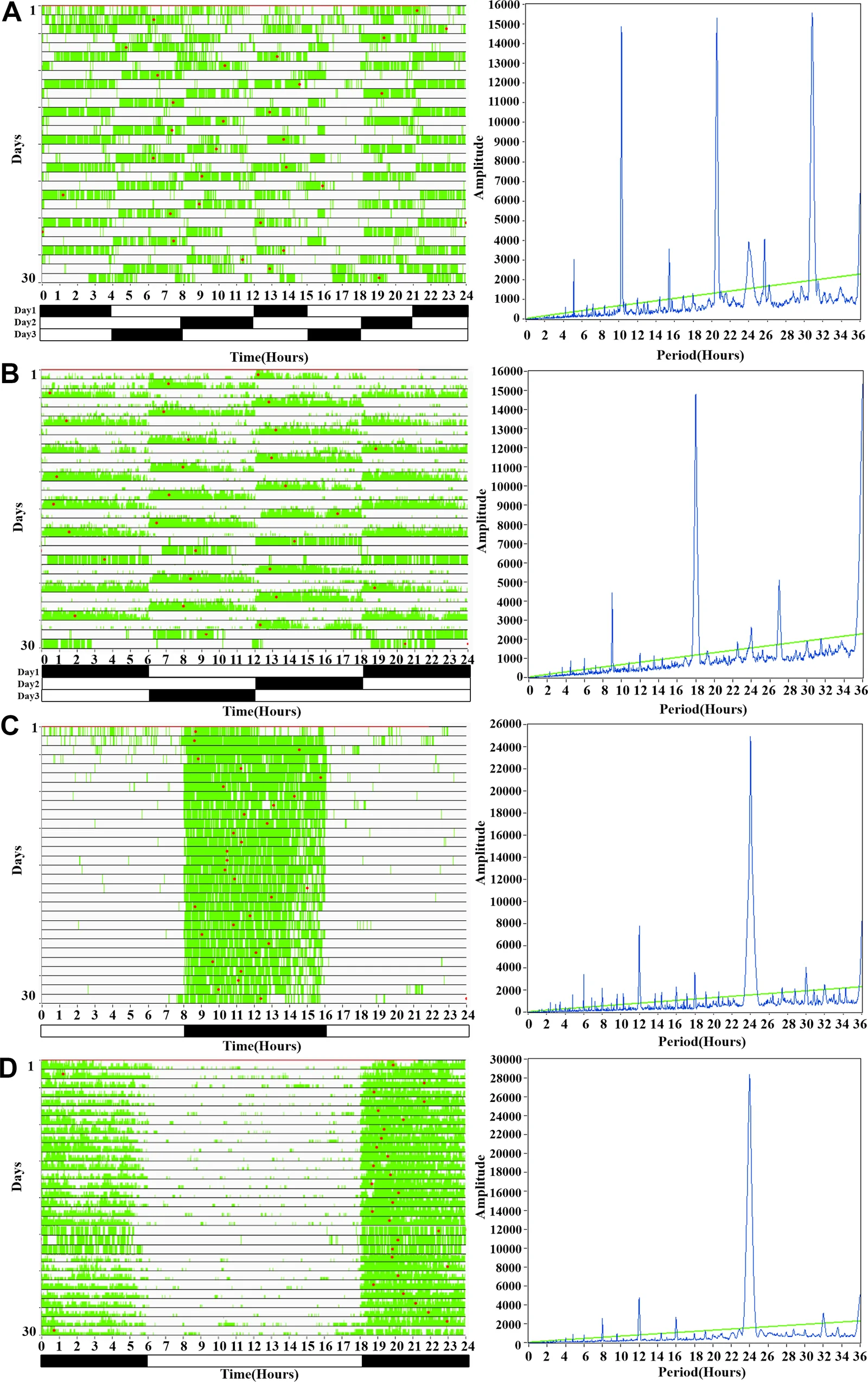
The actograms and chi-square periodogram analysis of locomotor activity rhythms for mice in different light/dark (LD) cycles [*group A* (*A*), *group B* (*B*), *group C* (*C*), and control group (*D*)]. *Left*: one representative actogram for mice exposed to the different LD cycles. Activity counts are indicated by the vertical green marks. The 30 rows on the *y*-axis represent the 30 days of the experimental period, and the *x*-axis represents the 24 h of a single day. The black areas at the *bottom* indicate the dark periods, whereas the white areas indicate the light periods. For *groups A* and *B*, because their LD cycle spans 3 days, the dark and light periods are shown across a 3-day interval. *Right*: one representative periodogram of locomotor activity rhythms for mice exposed to the different LD cycles. The *x*-axis represents the trial period (hours). The *y*-axis shows the chi-square value, which quantifies the rhythmic strength at each trial period. The green line marks the statistical significance threshold at α = 0.05. Any peak that rises above this green line corresponds to a statistically significant period.

**Figure 4.**
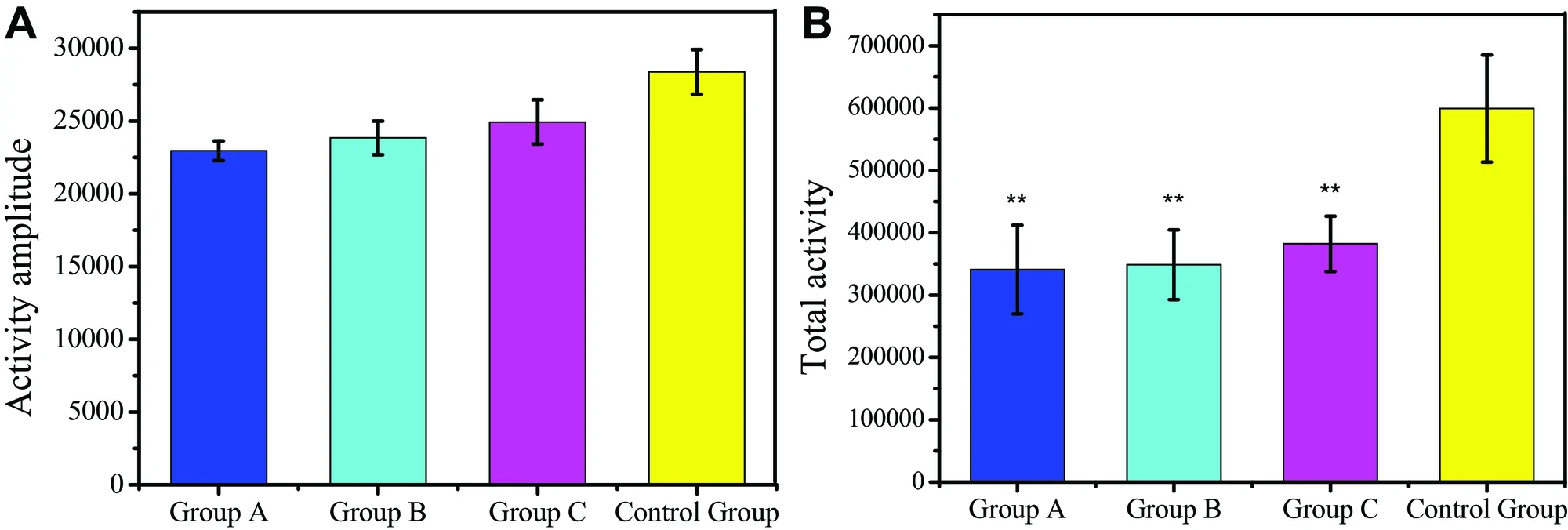
The rest-activity rhythm amplitude (*A*) and total activity (*B*) of different groups (data are presented as means ± SE. **P < 0.01 vs. control group; one-way ANOVA followed by Tukey’s post hoc test. *n* = 36 per group).

Representative actograms for each LD cycle condition are shown in Fig. 3. Activity counts are indicated by vertical green marks. The actigraphy data revealed fragmented activity patterns with multiple periodic components, consistent with unstable or partial entrainment rather than full adaptation to the imposed ultradian LD cycles. Spontaneous locomotor activity was observed during the dark period after 1 or 2 days, whereas little activity occurred during the light period. The circadian rhythm of rest-activity can be evaluated in terms of amplitude and period. The ClockLab analysis software provides a periodogram algorithm method to assess the circadian rhythm period of rest-activity. This method quantifies the variance of data across different periods relative to a 95% confidence interval (CI). In the periodogram, the green line indicates the chi-square significance threshold at α = 0.05. The dominant period was defined as the period length corresponding to the highest chi-square (Qp) value among all statistically significant peaks within a predefined search range (<36 h). The dominant rest-activity rhythm period, assessed via the periodogram method, corresponded to the respective LD cycle (Fig. 4). The presence of multiple significant periods (e.g., both ∼24-h and ∼9-h or ∼18-h components) was interpreted as direct evidence of incomplete entrainment and internal desynchronization, rather than stable adaptation to the imposed schedules. The dominant period therefore represents the strongest rhythmic component under these conditions, but does not imply full entrainment to the light-dark cycle. The dominant periods for *groups A*, *B*, *C*, and the control group were 10.28 h, 18 h, 24 h, and 24 h, respectively. Notably, although the disrupted LD cycles altered behavioral rhythms, activity amplitude did not differ between the control and experimental groups (Fig. 4*A*). Total activity level was higher in the control group than in the other groups (Fig. 4*B*), attributable to its longer dark period (12 h) compared with the other groups (8 h). These results indicate that, under conventional 24-h LD cycles, light serves as the principal zeitgeber for entraining rest-activity rhythms. However, under ultradian cycles (9-h or 18-h total period), the same light signals produce masking and internal desynchronization rather than stable entrainment.

### Effects of Shift Schedules on Behavioral Parameters in Mice

**Figure 5.**
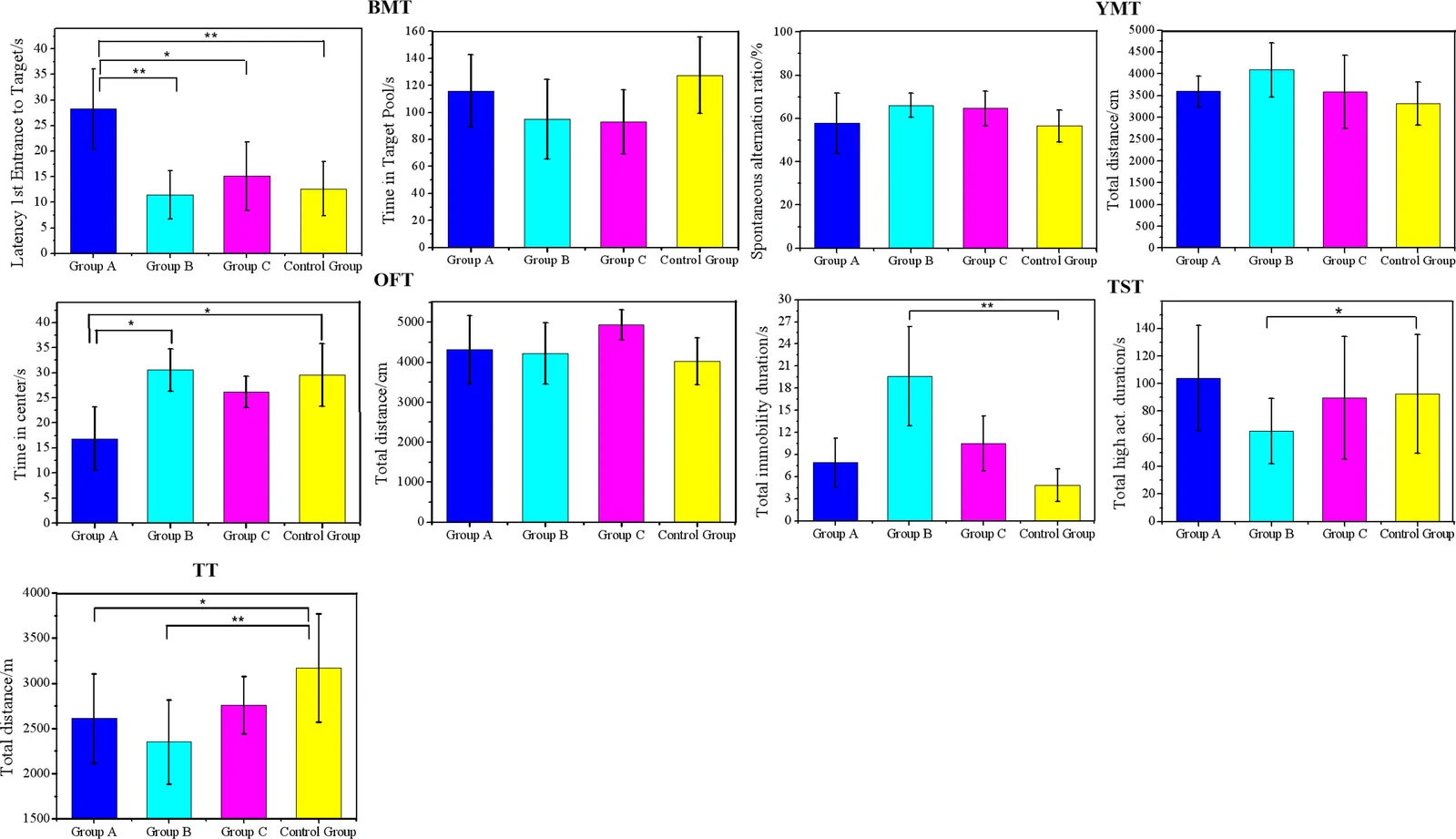
Effects of shift schedules on behavioral performance of mice. BMT: latency to first entry into the target hole and time spent in the target hole evaluated by the Barnes maze test; YMT: spontaneous alternation ratio and total distance traveled evaluated by the Y-maze test; OFT: time spent in the center of the arena and total distance traveled evaluated by the open field test; TST: total immobility duration and total high-activity duration evaluated by the tail suspension test; TT, total running distance evaluated by the treadmill test. (Data are presented as means ± SE. *P < 0.05, **P < 0.01; one-way ANOVA followed by Tukey’s post hoc test. *n* = 6 per group for BMT, YMT, OFT, and TST; *n* = 8 per group for TT.) BMT, Barnes maze test; OFT, open field test; TST, tail suspension test; TT, treadmill test; YMT, Y-maze test.

The effects of shift schedules on mood-related behaviors, memory, and fatigue were assessed through behavior tests. Spatial memory was assessed by using BMT. In the BMT, latency to first entry into the target hole was longer in *group A* than in the other groups (*P* < 0.05), whereas time spent in the target hole did not differ significantly among groups (Fig. 5, BMT). This suggests that the altered behavioral rhythm in *group A* impaired spatial memory. Short-term memory was evaluated using the YMT. No significant differences were observed in spontaneous alternation ratios or total distance traveled among groups (Fig. 5, YMT). Anxiety-like behavior was assessed via OFT. Mice in *group A* spent significantly less time in the center of the arena compared with the control group and *group B* (Fig. 5, OFT), indicating increased anxiety-like behavior. This reduction was not due to decreased locomotor activity, as total distance traveled during the OFT was similar across groups (Fig. 5, OFT). Depression-like behavior was examined using the TST. Mice in *group B* showed a significant increase in immobility time and a decrease in high-activity time compared with other groups (Fig. 5, TST), whereas no significant differences were observed among *group A*, *group C*, and the control group. These results indicate that non-24-h LD cycles can induce depression-like behavior. Overall, disruption of behavioral rhythm appears linked to the development of negative affective states such as anxiety and depression. Endurance and fatigue were evaluated using the TT. Total running distances were significantly lower in *groups A* and *B* (non-24-h LD cycles) compared with the control group (Fig. 5, TT), indicating reduced endurance and increased fatigue under non-24-h behavioral rhythms. Behavioral test results revealed that the altered light-dark (LD) cycles exerted a more pronounced impact on both behavioral and psychological measures in *groups A* and *B* compared with *group C*. This difference corresponded to the distinct shift schedules used: *groups A* and *B* underwent non-24-h rapid shift rotation, whereas *group C* maintained a fixed 24-h shift schedule. The speed of shift rotation significantly influences the synchronization of circadian rhythms. When shift rotation is slow (e.g., weekly or longer), the body has more time to gradually adjust to the new schedule, reducing circadian disruption. Conversely, rapid shift rotation (e.g., every 2–3 days) does not allow sufficient time for the circadian system to adapt, leading to persistent misalignment between internal rhythms and external time cues. This disruption can result in sleep fatigue and impaired cognitive function.

### Effects of Shift Schedules on Serum Hormones Rhythm in Mice

**Figure 6.**
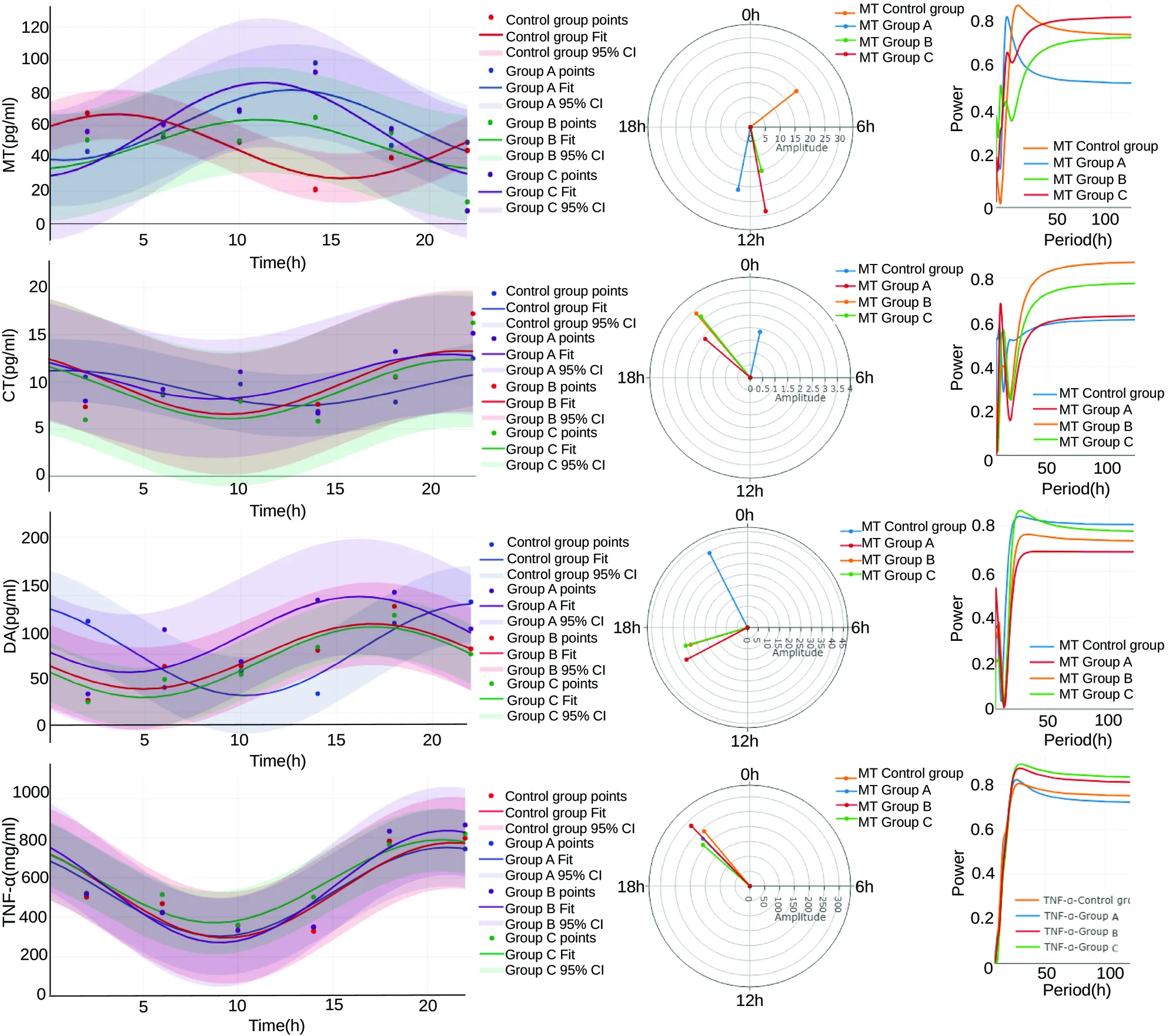
Cosinor fit plots of serum hormones (*n* = 6 per group). Shaded area represents 95% confidence interval of the fitted rhythm, based on amplitude CI (via delta method), not individual data point variability. All animals were transferred to constant darkness (DD) at the end of the final LD cycle (*time 0 h*). Samples were then collected at 2, 6, 10, 14, 18, and 22 h (every 4 h). The *x*-axis indicates hours after DD onset. CI, confidence interval; LD, light-dark.

The 24-h rhythmic data of serum hormones (MT, CT, DA, and TNF-α) were analyzed using a cosinor model. The rhythmic parameters are shown in Table 1, and the cosinor fit plots are shown in Fig. 6. Melatonin (MT) is a core circadian marker. In the control group, MT levels exhibited a significant 24-h rhythm (*P* < 0.05), with high secretion at dark phase and low levels during light phase, peaking at an acrophase of 3.47 h. However, this 24-h rhythm was absent in all other groups, and the peak phase showed a significant shift (Supplemental Fig. S1). The rhythmic mesor of MT was significantly higher in *group A* and *group C* compared with the control group (*P* < 0.05), which may be attributed to the shorter light exposure duration in the control group (12 h) relative to *groups A* and *C* (16 h). Cortisol (CT) is the primary glucocorticoid in rodents. It exhibits a robust diurnal rhythm that is tightly coupled to the light-dark cycle and the activity-rest cycle. However, the 24-h rhythm of CT was absent in all groups in this study, likely due to its high sensitivity to external disturbances (e.g., handling and blood sampling), which may mask its underlying rhythm. Dopamine (DA) is associated with movement and alertness. Its rhythmic secretion is closely associated with locomotor activity and wakefulness, making it relevant for assessing behavioral rhythm changes under altered LD cycles. A significant 24-h rhythm was observed in the control group and *group C* (both under 24-h LD cycles; *P* < 0.05), but was absent in *group A* and *group B* (non-24-h LD cycles; *P* > 0.05). In the control group, serum DA peaked at dark phase (22.20 h) and reached its nadir during light phase, whereas in *group C*, the phase was reversed, with a trough late at dark phase and a peak during light phase (16.91 h, Supplemental Fig. S1). Tumor necrosis factor-alpha (TNF-α) is a pleiotropic cytokine involved in inflammation and immune regulation. Circadian disruption is known to affect immune function. A ∼24-h rhythm was present in all groups, with higher levels at dark phase (acrophase ∼21 h) and lower levels during light phase.

**Table 1.** The cosinor fit parameters of serum hormones (n = 6 per group)

Hormones	Parameter	*Group A*	*Group B*	*Group C*	Control Group
MT	Mesor	60.20	48.39	57.44	47.29
	Amplitude	21.39	15.15	28.68	19.57
	Acrophase, h	12.74	11.05	11.31	3.47
	*P* value	0.197	0.432	0.213	0.045
CT	Mesor	10.55	9.92	9.22	9.35
	Amplitude	2.37	3.35	3.14	1.87
	Acrophase, h	20.71	21.33	21.41	0.82
	*P* value	0.555	0.377	0.472	0.331
DA	Mesor	116.90	98.10	93.21	103.77
	Amplitude	32.33	27.83	30.18	39.15
	Acrophase, h	16.15	16.87	16.91	22.20
	*P* value	0.261	0.184	0.041	0.041
TNF-α	Mesor	528.54	554.83	581.72	536.80
	Amplitude	223.81	282.22	209.18	239.66
	Acrophase, h	21.02	21.05	20.75	21.34
	*P* value	0.079	0.061	0.056	0.048

CT, cortisol; DA, dopamine; MT, melatonin; TNF-α, tumor necrosis factor alpha.

### Effects of Shift Schedules on Clock Genes Expression in the SCN of Mice

**Figure 7.**
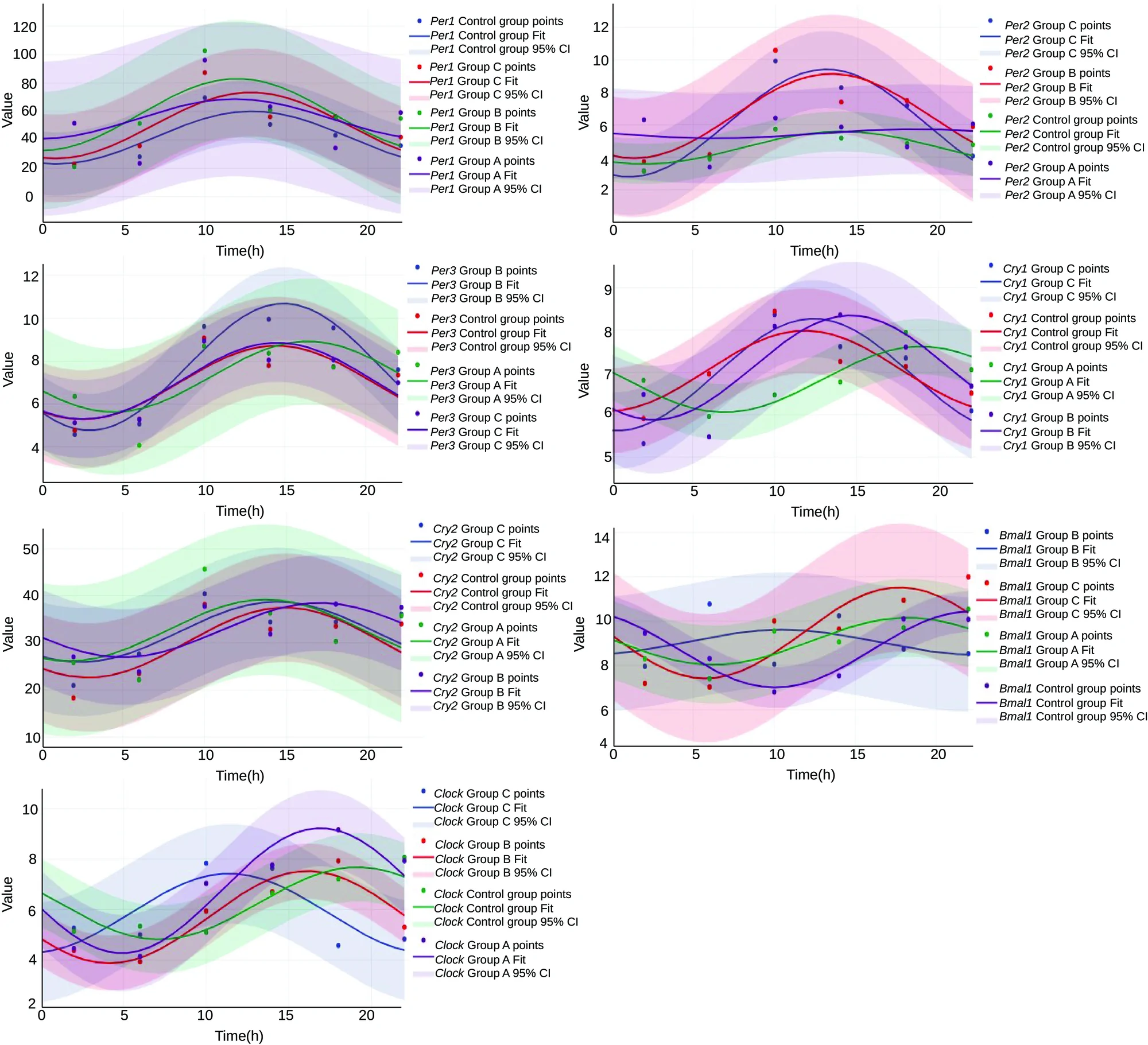
Cosinor fit plots of clock genes in SCN of mice (*n* = 6 per group). Shaded area represents 95% confidence interval of the fitted rhythm, based on amplitude CI (via delta method), not individual data point variability. All animals were transferred to constant darkness (DD) at the end of the final LD cycle (*time 0 h*). Samples were then collected at 2, 6, 10, 14, 18, and 22 h (every 4 h). The *x*-axis indicates hours after DD onset. LD, light-dark; SCN, suprachiasmatic nucleus.

The effects of shift schedules (LD cycles) on diurnal variation of clock gene expression in the SCN were examined through transcriptome analysis. Relative mRNA expression levels of the core clock genes measured every 4 h are shown in Fig. 7. In the core transcription-translation feedback loop of the mammalian circadian clock, the CLOCK and BMAL1 proteins form a heterodimer that acts as a positive regulator, binding to E-box elements to initiate the transcription of negative-feedback genes such as *period* (*Per1-3*) and *cryptochrome* (*Cry1-2*). The subsequently accumulated PER and CRY proteins then translocate into the nucleus and inhibit the activity of CLOCK-BMAL1, thereby establishing the molecular oscillatory basis that sustains circadian rhythms. Under 24-h LD cycles (control and *group C*), multiple clock genes in the mouse SCN exhibited robust circadian oscillations. In the control group, significant rhythms were detected for *Per1* (*P* = 0.044), *Cry1* (*P* = 0.047), and *Bmal1* (*P* = 0.022), and in *group C*, significant rhythms were present for *Per1* (*P* = 0.049), *Per2* (*P* = 0.048), *Per3* (*P* = 0.045), and *Cry1* (*P* = 0.043). In contrast, under non-24-h cycles (*groups A* and *B*), circadian rhythmicity was largely disrupted. In *group A*, none of the genes reached significance (all *P* > 0.05; *Clock P* = 0.061), and in *group B*, only *Per3* showed a significant oscillation (*P* = 0.027), whereas *Clock* exhibited a trend (*P* = 0.060) and *Bmal1* was clearly arrhythmic (*P* = 0.802). These *P* value patterns indicate that non-24-h light cycles abolished or severely dampened the significant circadian expression of most clock genes. The acrophases were markedly altered by the non-24-h conditions (Supplemental Fig. S2). Within the 24-h groups, *Bmal1* peaked at 22.08 h (control) and 17.75 h (*group C*), and *Per1*, *Per2*, and *Per3* peaked between 12.82 and 14.52 h. Under non-24-h cycles, large phase shifts occurred: the *Bmal1* acrophase advanced dramatically to 10.49 h in *group B*, whereas *Cry1* delayed from ∼12 h in the 24-h groups to 18.92 h in *group A*. Regarding amplitude, as shown in Supplemental Fig. S2, the negative-feedback genes *Per2* and *Cry1* reached their highest amplitudes under 24-h conditions in *group C* (3.30 and 1.32 h, respectively), whereas in non-24-h *group A*, *Per2* amplitude was nearly abolished (0.28 h). Similarly, *Bmal1* amplitude was reduced in non-24-h *groups A* and *B* (1.06 and 0.56 h) compared with the 24-h groups (1.71 and 2.05 h). Notably, *Clock* amplitude was highest in *group A* (2.47 h) but without reaching statistical rhythmicity. Collectively, these results demonstrate that non-24-h light cycles profoundly disrupt the coordinated circadian expression of clock genes in the SCN, as evidenced by the loss of significant rhythmicity, large phase desynchronizations, and dampened amplitudes of critical oscillators.

### Effects of Shift Schedules on Transcriptomic Profiles in the SCN of Mice

**Figure 8.**
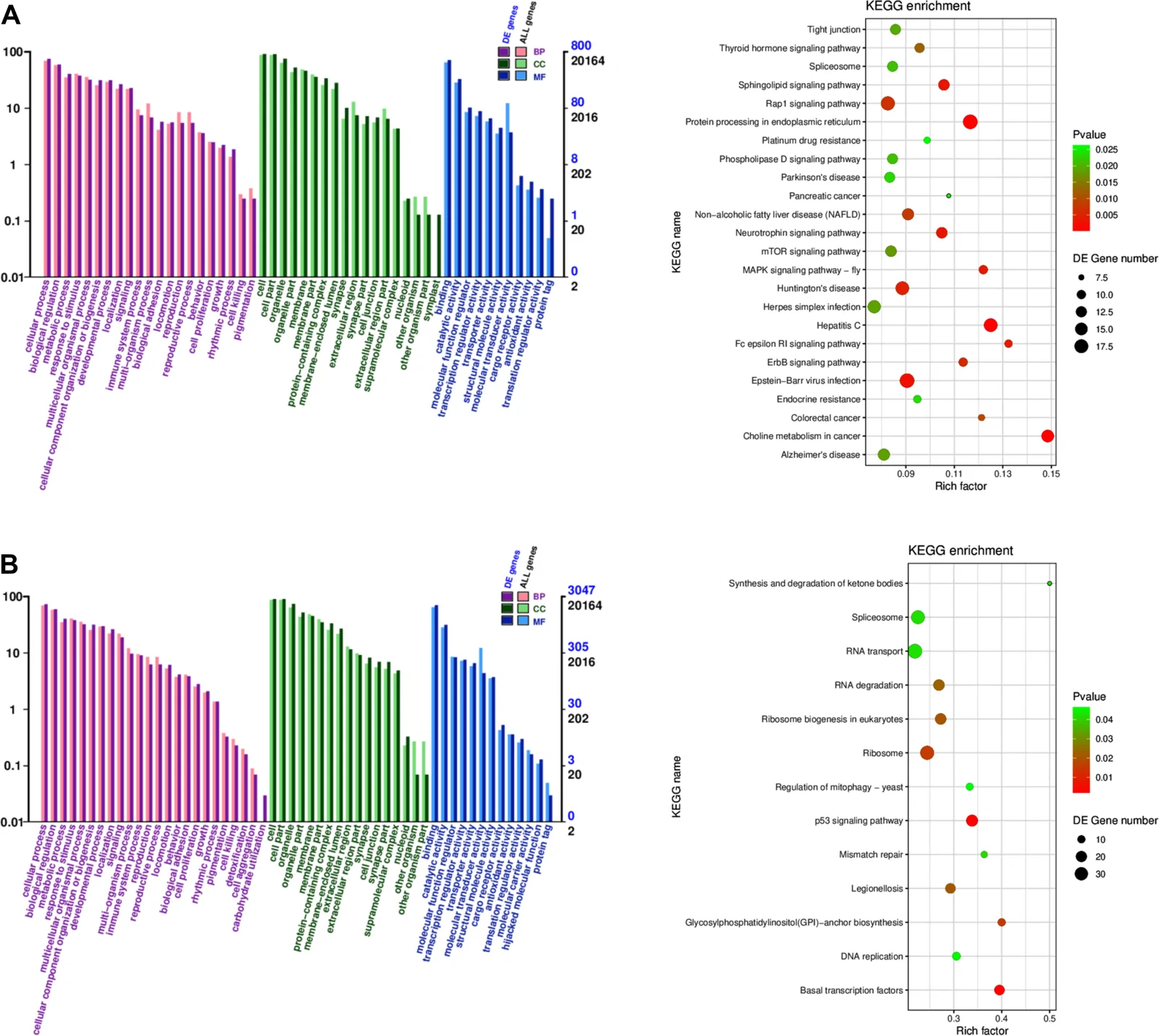
The GO and KEGG enrichment analyses of genes in the SCN of mice [*n* = 6 per group. All animals were transferred to constant darkness (DD) at the end of the final LD cycle (*time 0 h*). Samples were then collected at 2, 6, 10, 14, 18, and 22 h (every 4 h)]. *A*: GO and KEGG analyses of genes with rhythmic expression across all four groups. *B*: GO and KEGG analyses of genes without rhythmic expression across all four groups. GO, Gene Ontology; KEGG, Kyoto Encyclopedia of Genes and Genomes; LD, light-dark; SCN, suprachiasmatic nucleus.

To examine the effects of shift schedules on 24-h circadian gene expression in the SCN sampled at six time points (0200, 0600, 1000, 1400, 1800, and 2200) over a 24-h period. Total RNA of SCN was extracted, and RNA sequencing was performed. The gene expression rhythmicity was analyzed by cosinor fitting over a 23–25 h period. Analysis of circadian rhythms revealed that the expression of 932 genes in the SCN exhibited a 24-h rhythm across all four groups. GO and KEGG enrichment analyses results of these genes are shown in Fig. 8*A.* In GO analysis, cellular process, biological regulation, and metabolic process were the top categories. KEGG analysis revealed enrichment in pathways such as choline metabolism in cancer, Fc epsilon RI signaling pathway, and others related to cancer, allergy, and hepatitis C. As shown in Supplemental Fig. S3 (red boxes indicate enriched genes), in the choline metabolism pathway, PI3K/AKT, JNK, HIF1, and chaperonin containing TCP-1 were enriched, mediating overexpression of choline cycle enzymes and activation of the Ras-Raf-MAPK cascade. The Fc epsilon RI pathway showed enrichment and activation of PI3K/AKT, promoting inflammation. The hepatitis C pathway exhibited enrichment of interferon-stimulated genes [e.g., 2′-5′OAS, IRF-7, double‑stranded RNA‑activated protein kinase (PKR)], associated with antiviral responses.

GO and KEGG analyses were also performed on genes that did not show circadian expression rhythms in any of the four groups (Fig. 8*B*). Enriched GO terms included those related to metabolism, growth and development, circadian regulation, and cellular homeostasis. KEGG analysis revealed enrichment in synthesis and degradation of ketone bodies, basal transcription factors, and the p53 signaling pathway. Significantly enriched KEGG pathways are presented in Supplemental Fig. S4, with enriched genes highlighted in red boxes. Notably, the “Synthesis and degradation of ketone bodies” pathway included key rate-limiting enzymes: hydroxymethylglutaryl-CoA synthase (EC:2.3.3.10) for synthesis and 3-oxoacid CoA-transferase (EC:2.8.3.5) for degradation. These findings suggest that the enriched genes in this pathway may significantly influence obesity and mitochondrial respiratory chain function through ketone body metabolism. Furthermore, enrichment was observed in the “Basal transcription factors” pathway, including the core general transcription factors TFIIA, TFIIB, and TATA-box binding protein, which are essential for the assembly of the transcription preinitiation complex at gene promoters. In the “p53 signaling pathway,” key effector genes such as checkpoint kinase 1, p21 (CDKN1A), p53R2 (RRM2B), and growth arrest and DNA damage-inducible 45 (Gadd45) were enriched. p53R2 is a p53-inducible subunit of ribonucleotide reductase critical for DNA damage repair, whereas Gadd45 is involved in cell cycle control and DNA damage response. The enrichment of p21, a central mediator of p53-dependent cell cycle arrest, suggests the potential activation of this tumor-suppressive pathway. However, persistent dysregulation of the p53/p21 axis could, in certain contexts, contribute to altered cell fate, potentially influencing proliferation, metastasis, and cancer susceptibility.

We conducted GO and KEGG analyses on three gene sets, each consisting of genes that maintained circadian rhythmic expression in both a treatment group (*A*, *B*, or *C*) and the control group. GO analysis (Supplemental Fig. S5) revealed common enrichment in enzyme binding, cellular component biogenesis, mitochondrion, and protein-containing complex across groups. *Group B* specifically showed enrichment in SUMO polymer binding and mitochondrial respiratory chain complexes. *Group C* showed enrichment in intermediate filament binding and response to topologically incorrect protein, suggesting impaired protein repair. KEGG analysis (Supplemental Fig. S6) commonly enriched hepatitis C, choline metabolism in cancer, and nonalcoholic fatty liver disease. *Group A* specifically showed enrichment in endometrial cancer, forkhead box O signaling, and epidermal growth factor receptor (EGFR) signaling pathways. *Group B* showed specific enrichment in Huntington’s disease, Alzheimer’s disease, and Parkinson’s disease pathways.

Finally, GO and KEGG analyses were performed on genes that lost circadian rhythmicity in the experimental groups (*A*, *B*, and *C*) compared with the control group (Supplemental Figs. S7 and S8). In *group A*, GO terms related to transcription initiation/repression, synaptic vesicles, and intercellular signaling were enriched; KEGG terms included glycosphingolipid biosynthesis and cell cycle. In *group B*, GO terms included protein binding, endocytosis, and signal transduction; KEGG terms included glycosphingolipid biosynthesis, glycolysis/gluconeogenesis, estrogen signaling, alcoholism, and viral carcinogenesis. In *group C*, GO terms included kinase inhibitor activity, synapses, and cellular signaling; KEGG terms included acute myeloid leukemia, vasopressin-regulated water reabsorption, and estrogen signaling.

In summary, genes that lost rhythmicity in experimental groups were enriched in metabolic and regulatory pathways such as glycolysis/gluconeogenesis and estrogen signaling. Altered LD cycles disrupted endocrine and metabolic rhythms, compromising health in *groups A*, *B*, and *C*. Enrichment of pathways related to basal cell carcinoma, viral carcinogenesis, and acute myeloid leukemia suggests that shift work may elevate cancer risk via circadian disruption.

### Effects of Shift Schedules on Gut Microbiome in Mice

**Figure 9.**
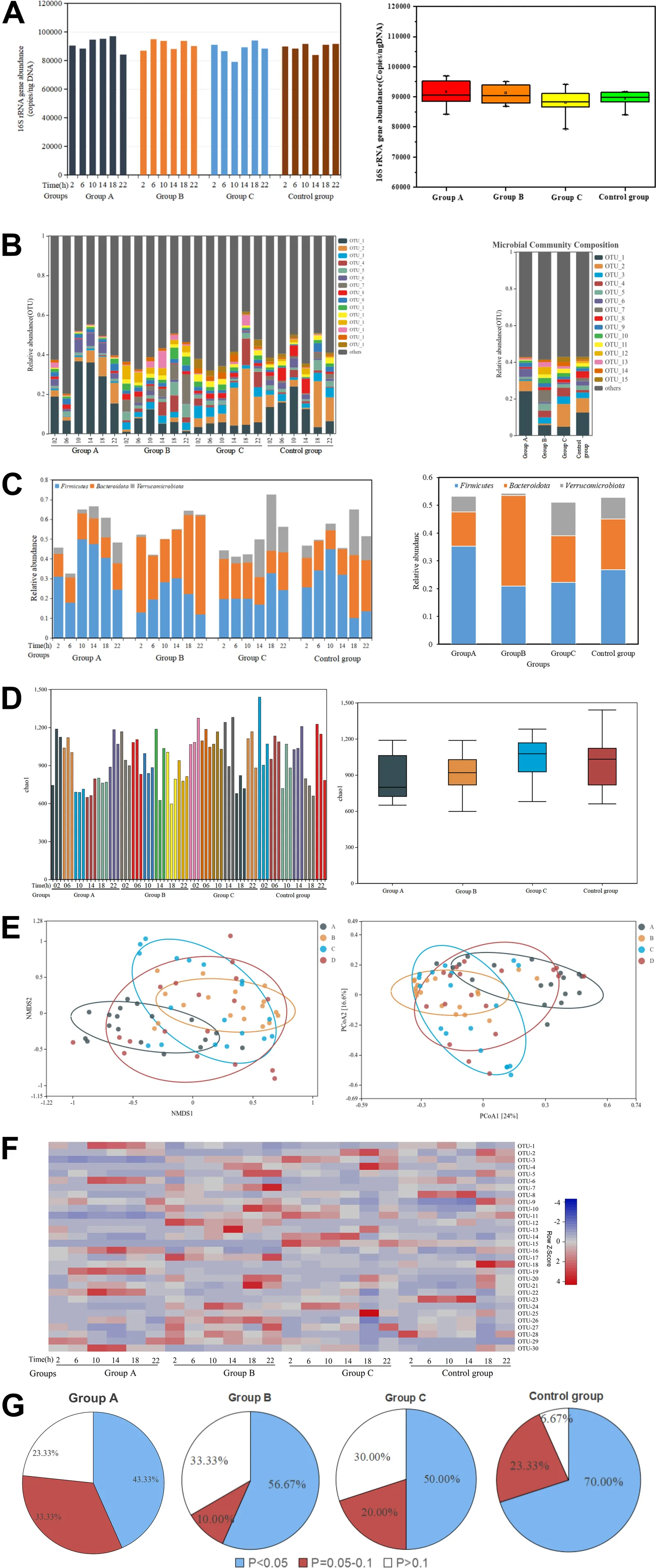
Shift schedules alter gut microbial community composition and oscillations (*n* = 6 mice per treatment per time point, *n* = 36 per group). All animals were transferred to constant darkness (DD) at the end of the final LD cycle (*time 0 h*). Samples were then collected at 2, 6, 10, 14, 18, and 22 h (every 4 h). The *x*-axis indicates hours after DD onset. *A*: average absolute 16S rRNA gene copy number across time points and groups. *B*: relative abundances of dominant OTUs averaged across time points and groups. *C*: relative abundances of the three main phyla averaged across time points and groups. *D*: alpha diversity (Chao1 index). *E*: beta diversity (NMDS and Bray–Curtis PCoA) analyses. *F*: heatmap of 16S rRNA relative abundances (columns: OTUs and rows: samples). *G*: proportions of oscillating and nonoscillating OTUs determined via cosinor fit (period length: 23 h–25 h). LD, light-dark; OTU, operational taxonomic unit.

We analyzed microbial community composition and diversity in fecal samples collected every 4 h via 16S rRNA gene amplicon sequencing (QIIME pipeline). Average 16S rRNA gene copy numbers did not differ among groups (Fig. 9*A*). For comparison with traditional taxonomy-based approaches, OTUs were also generated by clustering sequences at 97% pairwise identity using UPARSE. An operational taxonomic unit (OTU) is defined as a cluster of 16S rRNA gene sequences sharing ≥97% sequence similarity, serving as a proxy for a microbial species in subsequent diversity and composition analyses. The top 15 OTUs, with relative abundances above 1% were used to draw relative abundance distributions across groups and time points (Fig. 9*B*). Among the top 30 OTUs (Supplemental Table S2), there are three main phyla: Firmicutes, Bacteroidota, and Verrucomicrobia at the phylum level (Fig. 9*C*). Firmicutes was the most abundant phylum in all groups. Its relative abundance was significantly higher in *group A* than in the control group (*P* < 0.05), whereas no significant differences in Firmicutes abundance were observed between *group B* and the control group (*P* > 0.05). Bacteroidetes abundance was significantly lower in *group A* compared with control group (*P* < 0.05), but significantly higher in *group B* compared with control group (*P* < 0.05). Verrucomicrobia abundance was significantly reduced in both *group A* and *group B* relative to control (*P* < 0.05). *Group A* had a significantly higher Firmicutes-to-Bacteroidota (F/B) ratio than the control group (*P* < 0.05). In contrast, *group B* exhibited a significantly lower F/B ratio compared with control (*P* < 0.05), driven by its increased Bacteroidetes abundance. *Group C*, which underwent a fixed 24-h shift schedule, showed no significant differences in Firmicutes, Bacteroidetes, Verrucomicrobia abundances, or F/B ratio compared with the control group, indicating that a 24-h shifted cycle did not induce the same compositional shifts as the non-24-h ultradian cycles. The compositional changes observed in *group A* (higher Firmicutes, lower Bacteroidetes, elevated F/B ratio) are consistent with previous reports of gut microbiota alterations under circadian disruption, including rotational night shift work in humans (11) and chronic jet lag in mice (12). However, *group B* showed a distinct pattern (higher Bacteroidetes, lower F/B ratio), indicating that different LD schedules can induce divergent microbiota responses. The reduced Verrucomicrobia in both *group A* and *group B* suggests a common sensitivity of this phylum to altered light-dark cycles. Alpha diversity (Chao1 index) varied over time and was significantly lower in *groups A* and *B* (Fig. 9*D*; *P* < 0.05). The nonmetric multidimensional scaling (NMDS) and PCoA plots revealed a clear separation between the microbial communities of the control and treatment groups (Fig. 9*E*). As shown in Table 2, PERMANOVA confirmed a significant difference in overall community composition among groups (*R*^2^ = 0.222, *P* = 0.001). Subsequent pairwise comparisons further revealed significant differences between control group and *group A* (*R*^2^ = 0.14, *P* = 0.018), between control group and *group B* (*R*^2^ = 0.149, *P* = 0.001), and between control group and *group C* (*R*^2^ = 0.075, *P* = 0.008).

**Table 2.** Analysis of significant differences in microbial community composition among groups (n = 36 per group)

Sub-condition 1 vs. Sub-condition 2	Df	Sums of Sqs	Mean Sqs	F.Model	*R* ^2^	Pr(>*F*)
*Group A* vs. *group B*	1	1.89	1.89	11.60	0.254	1e-03
*Group A* vs. *group C*	1	1.47	1.47	7.58	0.182	1e-03
*Group A* vs. *group D*	1	1.13	1.13	5.52	0.140	1e-03
*Group B* vs. *group C*	1	1.32	1.32	6.94	0.170	1e-03
*Group B* vs. *group D*	1	1.20	1.20	5.96	0.149	1e-03
*Group C* vs. *group D*	1	0.64	0.64	2.76	0.075	8e-03
All_groups	3	3.83	1.28	6.46	0.222	1e-03

Differences in microbial community composition among groups were assessed by PERMANOVA (adonis, 999 permutations) based on Bray–Curtis distance, and pairwise PERMANOVA with Benjamini–Hochberg false discovery rate correction was performed for post hoc comparisons. Df: degrees of freedom; FDR, false discovery rate; Mean Sqs, mean squares; Sums of Sqs, sum of squares; F.Model: F-statistic (model); R^2^: R-squared; Pr(>F): *P* value (Permutational).

The top 30 OTUs (Supplemental Table S2) were selected to generate a relative abundance heatmap across groups and time points (Fig. 9*F*). Their diurnal oscillations were assessed by cosinor fitting over a 23–25 h period (Fig. 9*G*). In the control group, 70% (21/30) of OTUs oscillated significantly (*P* < 0.05), and only 6.67% (2/30) had *P* > 0.1. By contrast, the proportion of oscillating OTUs was numerically lower in the experimental groups: 43.33% in *group A*, 56.67% in *group B*, and 50% in *group C*, accompanied by an increase in nonoscillating OTUs (*P* > 0.1). Although the proportion was numerically lower in the experimental groups, the differences did not reach statistical significance (chi-square test, χ^2^ = 4.71, df = 3, *P* = 0.194). Only four OTUs (OTU_19, 20, 21, and 29, assigned to Streptococcaceae, Muribaculaceae, and Lachnospiraceae) maintained significant oscillations across all groups, albeit with different abundances and acrophases. Cosinor analysis of the three main phyla (Supplemental Fig. S9) revealed that Firmicutes and Bacteroidetes exhibited a 24-h rhythm (*P* < 0.05) in the control group and *group B*, but not in *groups A* and *C*. Verrucomicrobia showed a 24-h rhythm in the control group and *group C*, but not in *groups A* and *B*. The substantial reduction in the number of rhythmic microbial taxa and the loss of 24-h periodicity in specific phyla indicate a disruption of the host-microbiome circadian dialogue. In this study, mice had ad libitum access to food, and the only experimental manipulation was the alteration of LD cycles. The disrupted LD schedules fragmented rest-activity rhythms and desynchronized the central circadian clock, which likely impaired the normal entrainment of peripheral gut clocks. Consequently, the loss of synchrony between the central clock and the gut microbiota led to reduced microbial oscillations. These results are consistent with previous reports that circadian disruption alone (e.g., chronic jet lag or shift-work schedules) can desynchronize peripheral clocks and dampen microbial rhythmicity even under constant feeding conditions (13, 14).

## DISCUSSION

In today’s 24/7 society, shift work has become an essential part of workers in hospitals, manufactory, and transport, especially of military field, with specific shift schedules-such as rapid rotation, slow rotation, 24-h, and non-24-h schedules-tailored to diverse mission environments. Each schedule exerts distinct physiological pressures by differentially disrupting the body’s circadian rhythms (3). This disruption can lead to varying degrees of health impairment (15), cognitive degradation (e.g., reduced vigilance and slowed reaction times), and affective dysregulation (e.g., increased anxiety; 16, 17). Therefore, systematically investigating the health impact of these distinct shift schedules on personnel is essential and imperative. Such research provides the critical evidence base required to develop optimized schedules and proactively protect shift worker health, sustain cognitive performance, and maintain emotional resilience, thereby directly supporting long-term occupational health and work performance.

Our study demonstrates that chronic exposure to disruptive LD cycles, simulating shift-work schedules, induces significant body weight gain in mice. The body weight data (Fig. 2) provide a fundamental phenotypic readout of metabolic disturbance, consistent with evidence that circadian disruption can promote weight gain and metabolic imbalance (4, 18). Although total activity levels were influenced by the duration of the dark period (Fig. 4*B*), the observed weight changes cannot be simply attributed to differences in overall activity, suggesting underlying metabolic dysfunction. This outcome aligns with a model wherein circadian misalignment dismantles the temporal architecture that normally coordinates energy intake, storage, and expenditure (18). The foundational deficit appears to be the functional disorganization of the SCN pacemaker. The ablation or severe distortion of master clock gene (*Per*, *Cry*, *Bmal1*) and circadian rhythms in the experimental groups, as shown in Fig. 7 and Table 3, particularly under non-24-h cycles, signifies a loss of the master coordinator. Transcriptomic analysis further revealed that genes losing rhythmicity were enriched in metabolic and signaling pathways (Fig. 8), a pattern consistent with findings that clock dysfunction in metabolic tissues is linked to obesity-related inflammation (19). This suggests that the SCN’s transcriptional output may shift toward a dysregulated metabolic state under disruptive schedules.

**Table 3.** The cosinor fit parameters of clock genes expression (n = 6 per group)

Genes	Parameter	*Group A*	*Group B*	*Group C*	Control Group
*Per1*	Mesor	18.68	19.44	17.54	15.41
	Amplitude	3.49	6.32	5.81	4.63
	Acrophase, h	11.82	11.98	12.82	12.88
	*P* value	0.739	0.094	0.049	0.044
*Per2*	Mesor	5.44	6.53	6.10	4.58
	Amplitude	0.28	2.59	3.30	1.00
	Acrophase, h	18.39	13.41	13.10	14.00
	*P* value	0.950	0.224	0.048	0.171
*Per3*	Mesor	7.27	7.73	7.08	7.00
	Amplitude	1.65	2.96	1.78	1.72
	Acrophase, h	16.41	14.92	14.52	14.47
	*P* value	0.339	0.027	0.045	0.203
*Cry1*	Mesor	6.84	7.11	6.95	7.04
	Amplitude	0.78	1.23	1.32	0.95
	Acrophase, h	18.92	14.63	12.37	11.84
	*P* value	0.073	0.118	0.043	0.047
*Cry2*	Mesor	32.70	32.74	32.35	30.08
	Amplitude	6.54	5.76	6.41	7.43
	Acrophase, h	13.71	17.02	14.43	14.90
	*P* value	0.51091	0.332	0.242	0.168
*Bmal1*	Mesor	9.09	9.05	9.47	8.71
	Amplitude	1.06	0.56	2.05	1.71
	Acrophase, h	18.23	10.49	17.75	22.08
	*P* value	0.303	0.802	0.226	0.022
*Clock*	Mesor	6.75	5.70	5.85	6.21
	Amplitude	2.47	1.82	1.57	1.38
	Acrophase, h	16.91	16.14	11.40	19.03
	*P* value	0.061	0.060	0.180	0.082

Locomotor activity analysis revealed that rest-activity rhythm rapidly aligned with the LD cycle within 1 to 2 days (Fig. 3). This rapid realignment underscores a compartmentalized response of the circadian system, where behavior can be quickly driven by light via direct retinohypothalamic tract-mediated SCN phase resetting (20, 21), even as molecular and endocrine rhythms remain disrupted. However, this superficial behavioral adaptation masks a deeper, more protracted internal desynchronization. Peripheral oscillators in metabolic tissues and the gut microbiome rely on secondary zeitgebers (e.g., feeding-fasting cycles, body temperature) that shift more slowly and can become uncoupled from the centrally driven activity rhythm (13, 14). This dissociation between rapidly entrained behavior and lagging internal physiology creates a state of metabolic and endocrine misalignment, constituting a core pathophysiological basis for the observed health deficits despite apparent behavioral adaptation. Importantly, the presence of multiple significant periodic components (e.g., ∼24-h together with ∼9-h or ∼18-h rhythms) in the ultradian LD groups (Fig. 3) indicates a state of incomplete and unstable entrainment rather than stable adaptation. Therefore, our findings should be interpreted as evidence that non-24-h schedules produce circadian disruption and internal desynchronization, which may model certain features of rapidly rotating shift work in humans.

Our behavioral findings revealed a selective vulnerability where spatial and short-term memory remained largely intact, whereas anxiety- and depression-like behaviors were significantly exacerbated in *groups A* and *B* (Fig. 5, OFT, TST). This pattern is clinically significant and consistent with reports of a higher prevalence of mood disorders over cognitive complaints in shift-working populations (6, 16). Mechanistically, this selective affective impairment can be understood as a pathogenic convergence of multisystem signals. The primary insult originates in the SCN, where the circadian expression rhythms of core clock genes were completely abolished (Fig. 7). This central disruption was paralleled by the loss of circadian dopamine (DA) rhythm in these same groups (Fig. 6 and Table 1). In contrast, *group C* and the control group maintained a significant DA rhythm and exhibited no significant depression-like behavior, indicating that stable neurochemical rhythmicity is crucial for emotional homeostasis. This is consistent with the established role of dopaminergic signaling in circadian entrainment and mood regulation (22). Concurrently, the gut dysbiosis we documented-characterized by altered F/B ratios and reduced Verrucomicrobia-represents a microbial signature that has been associated with impaired gut barrier function (23), which can predispose to low-grade endotoxemia. Such chronic inflammation could potentially affect the brain, as studies have linked gut microbiota disruption to neuroinflammation and depressive-like behaviors via the gut-brain axis (24, 25). Thus, the convergence of central neurochemical desynchronization and peripheral inflammatory signals may provide a biological basis for the mood disturbances prevalent in shift-work populations.

A parallel and likely interacting pathological cascade unfolds in the gut, as depicted comprehensively in Fig. 9. Our 16S rRNA sequencing data revealed that *groups A* and *B* developed distinct compositional shifts, characterized by altered relative abundances of Firmicutes, Bacteroidota, and Verrucomicrobia (Fig. 9*C*). Notably, *group A* exhibited an elevated Firmicutes/Bacteroidota (F/B) ratio, whereas *group B* showed a contrasting decrease, indicating that different shift schedules can elicit divergent microbiota responses. Critically, a key functional consequence was the profound loss of diurnal rhythmicity in specific gut microbial taxa. Although the decrease in the proportion of oscillating OTUs did not reach statistical significance (Fig. 9*F*), rhythmicity analysis at the phylum level revealed that non-24-h LD cycles selectively abolished the 24-h rhythms of dominant phyla-Firmicutes, Bacteroidetes, and Verrucomicrobia-in a schedule-dependent manner (Fig. 9*H*). The breakdown of this transkingdom temporal coordination, where beneficial microbial functions are no longer temporally aligned with host physiology, has been implicated in driving metabolic inefficiency (11). Although we did not directly measure gut permeability or systemic inflammation, the observed loss of microbial oscillations at the phylum level provides evidence of a disrupted host-microbe dialogue, a phenomenon that has been reported in clinical studies of shift workers (11, 26) and is associated with gastrointestinal and metabolic disturbances. We acknowledge that unmonitored changes in feeding patterns could have contributed to these gut microbial alterations. However, the concurrent loss of circadian rhythms in SCN clock genes and in key circadian hormones such as melatonin and dopamine indicate a direct, light-driven central disruption. Moreover, prior work has demonstrated that central circadian desynchronization alone can abolish gut microbial oscillations even when feeding is controlled (13), supporting a top-down disintegration of circadian organization rather than a phenomenon solely secondary to altered feeding.

The integrated analysis across behavioral, endocrine, transcriptional, and microbial levels reveals a hierarchical disintegration of circadian organization induced by non-24-h shift schedules. Although rest-activity rhythms rapidly re-entrained to new LD cycles, this superficial behavioral adaptation masked a state of internal desynchronization spanning from the central brain to the gut microbiome. The primary disruption occurred within the SCN. In *groups A* and *B*, the significant 24-h rhythms of core clock genes (*Per1*, *Cry1*, *Bmal1*) observed in the control group were no longer detected (Fig. 7 and Table 3), indicating a loss of coordinated circadian expression rather than stable entrainment. This functional disorganization was further evidenced by the decoupling of PER1 and BMAL1: under non-24-h cycles, BMAL1 rhythmicity was lost (*P* > 0.05), whereas PER1 retained only residual peak-like behavior, consistent with a breakdown of the classic antiphasic relationship (21). This SCN disorganization was accompanied by the loss of the 24-h melatonin rhythm in all experimental groups (Fig. 6 and Table 1), a hormone whose rhythmic secretion is directly regulated by SCN output (27). Similarly, the circadian rhythm of DA was lost in *groups A* and *B* but preserved in *group C* under a stable 24-h schedule, supporting the interpretation that rapid schedule rotation disrupts hormonal rhythmicity, whereas a fixed schedule permits coherent, albeit phase-shifted, rhythms (22). Transcriptomic analysis revealed that genes losing circadian rhythmicity were enriched in pathways governing metabolism (glycolysis/gluconeogenesis), cell signaling (estrogen), and cancer-related processes (p53 signaling) (Fig. 8, *G*–*I*), a pattern suggesting that circadian disruption may alter the transcriptional output of the SCN toward pathways associated with disease susceptibility (28, 29). Concurrently, in the gut ecosystem, non-24-h LD cycles selectively abolished the 24-h rhythms of dominant bacterial phyla, including Firmicutes, Bacteroidetes, and Verrucomicrobia (Fig. 9*G*). This observation aligns with previous reports that circadian disruption can desynchronize the gut microbiome, likely through the loss of coherent host-derived timing cues (30, 31).

In summary, our findings suggest a self-reinforcing pathological cascade driven by circadian disruption. Aberrant light cues disrupted the coordinated expression of core clock genes in the SCN, which was accompanied by the loss of circadian rhythmicity in neuroendocrine outputs, including melatonin and dopamine. This hormonal dysregulation, together with mistimed activity, was associated with gut microbiota dysbiosis and a loss of microbial diurnal rhythmicity at the phylum level. Given that gut dysbiosis has been linked to systemic inflammation and metabolic dysfunction in previous studies (23, 32), these multisystem changes may constitute the core pathophysiology linking shift work to its adverse health outcomes, including metabolic disorder, affective impairment, and increased disease risk. Consequently, clinical interventions for shift workers may need to extend beyond managing single symptoms. Strategies targeting the restoration of system-wide circadian harmony, such as circadian-informed scheduling, timed dietary interventions (33), and chronobiotic approaches aimed at the gut-brain axis, warrant further investigation. We acknowledge several limitations. Food intake was not monitored, so feeding-related metabolic effects cannot be ruled out, though the central circadian disruption and uniform feeding conditions support a circadian-driven basis. In addition, only male mice were used, which limits the generalizability of our findings given known sex differences in circadian regulation. However, the shift schedules modeled here are derived from military operational scenarios where personnel are predominantly male. Future studies should include female mice to test the model across sexes.

## Supplementary Material

Supplemental Material

Supplemental Material

Supplemental Material

Supplemental Material

Supplemental Material

Supplemental Material

Supplemental Material

Supplemental Material

Supplemental Material

Supplemental Material

Supplemental Material

## Data Availability

The data that support the findings of this article cannot be made publicly available due to the confidentiality policy of the authors' institution. The raw data are available from the corresponding author (Qun Luo) upon reasonable request and through collaborative investigations. All other data needed to evaluate the conclusions in the paper are presented in the paper or the supplementary materials.
