# Supplementary material for: Simulated shift work schedules disrupts circadian rhythms and behavior in mice
Source: Function (Oxf). 2026 Jul 10;7(5):e012-2026. doi: 10.1152/function.012.2026 (PMC13581034; doi:10.1152/function.012.2026)
Supplement: Supplementary file 1 [file function.012.2026_supplementary_figures1.pdf]

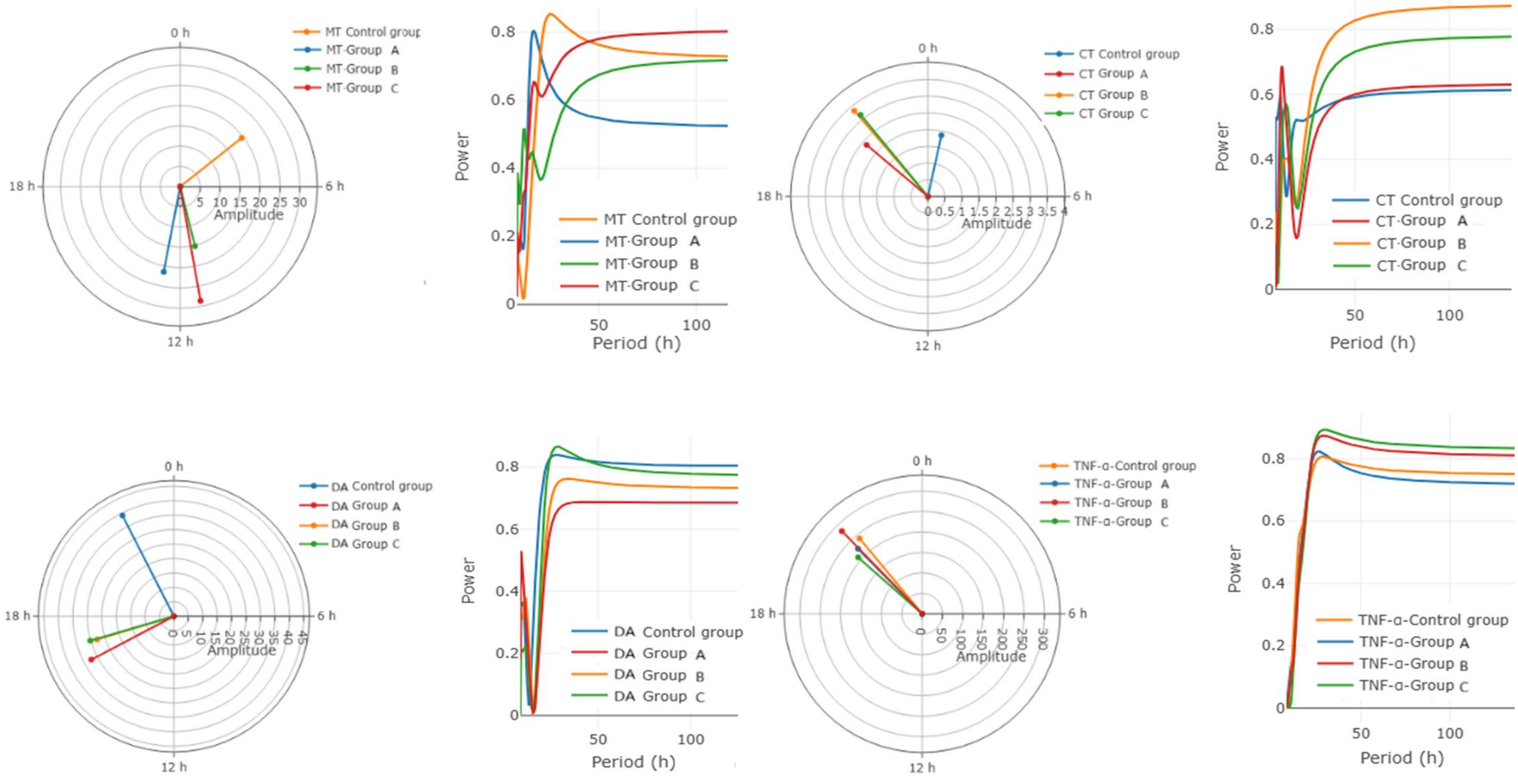

Polor plot Lomb-Scargle periodogram Polor plot Lomb-Scargle periodogram

Supplementary Fig.S1: Polor plot and Lomb-Scargle periodogram of serum hormones (n = 6 per group. Shaded area represents 95% confidence interval of the fitted rhythm, based on amplitude CI (via delta method), not individual data point variability. All animals were transferred to constant darkness (DD) at the end of the final LD cycle (time 0 h). Samples were then collected at 2, 6, 10, 14, 18, and 22 h (every 4 h). The x-axis indicates hours after DD onset.)
