# Supplementary material for: Simulated shift work schedules disrupts circadian rhythms and behavior in mice
Source: Function (Oxf). 2026 Jul 10;7(5):e012-2026. doi: 10.1152/function.012.2026 (PMC13581034; doi:10.1152/function.012.2026)
Supplement: Supplementary file 3 [file function.012.2026_supplementary_figures3.pdf]

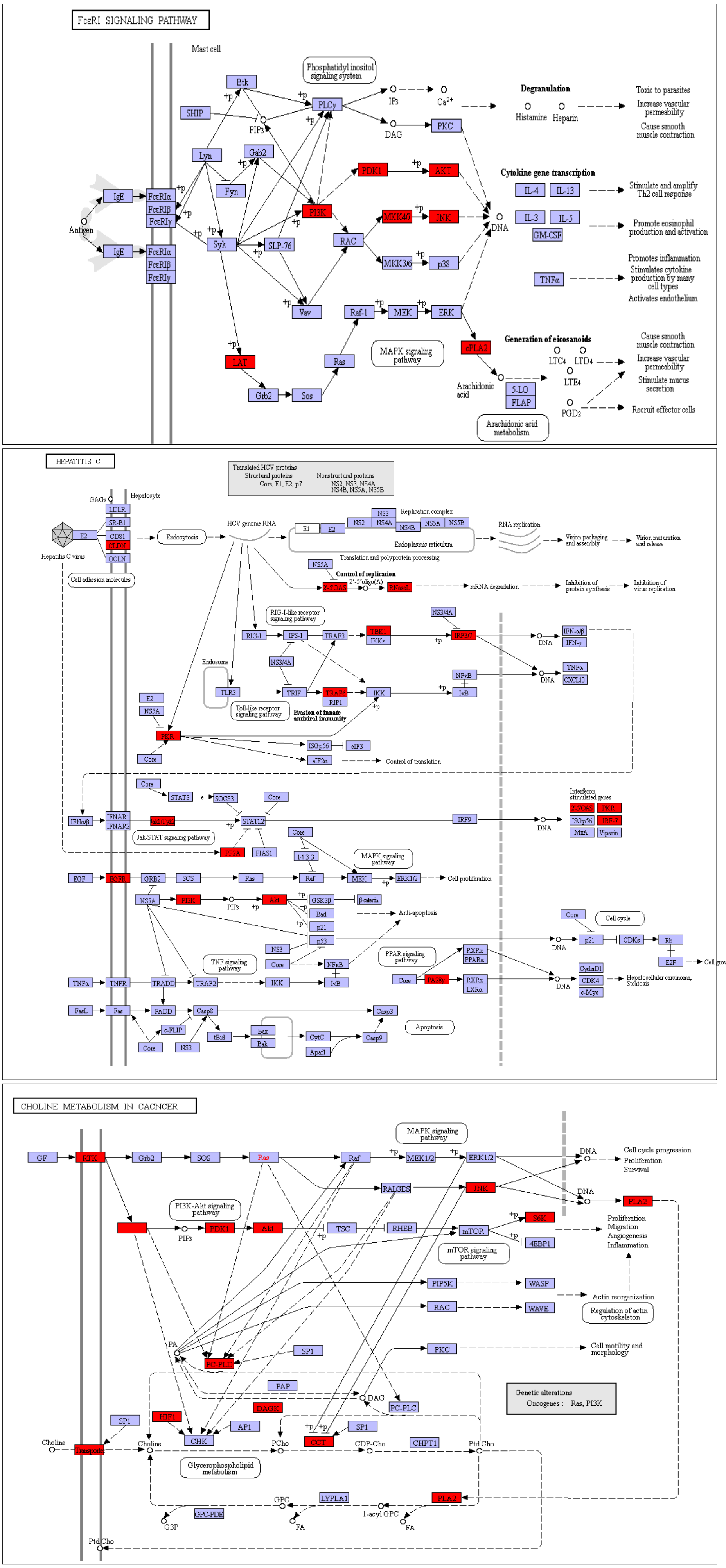

Supplementary Fig.S3: Key enriched KEGG pathways of genes with rhythmic expression in the SCN of mice across all four groups (n = 6 per group. All animals were transferred to constant darkness (DD) at the end of the final LD cycle (time 0 h). Sampling was performed every 4 h thereafter. Red boxes are enriched genes)
