# Supplementary material for: Simulated shift work schedules disrupts circadian rhythms and behavior in mice
Source: Function (Oxf). 2026 Jul 10;7(5):e012-2026. doi: 10.1152/function.012.2026 (PMC13581034; doi:10.1152/function.012.2026)
Supplement: Supplementary file 9 [file function.012.2026_supplementary_figures9.pdf]

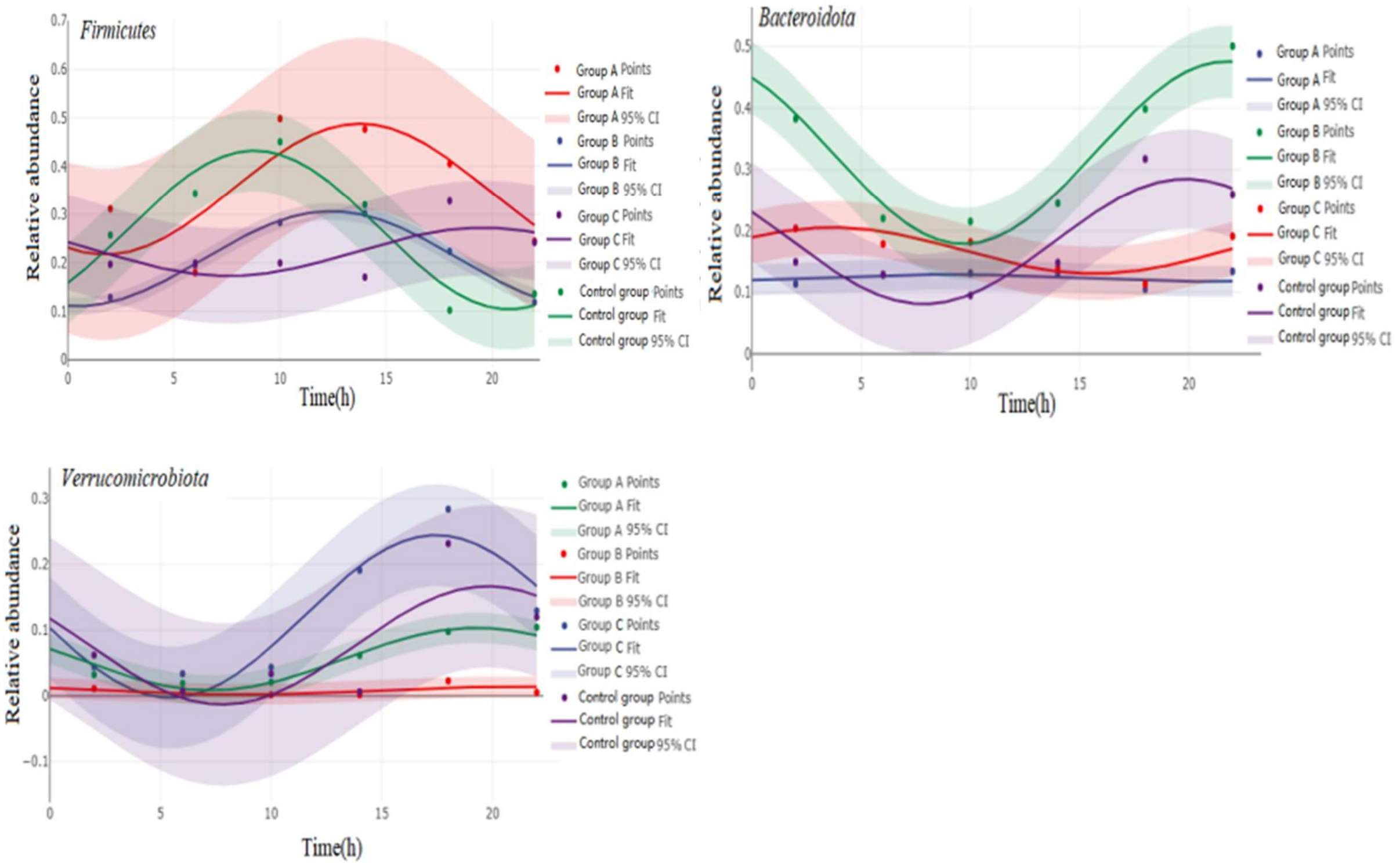

Supplementary Fig.S9: Cosinor fit plot for the three main phyla (n = 6 mice per treatment per time point. All animals were transferred to constant darkness (DD) at the end of the final LD cycle (time 0 h). Samples were then collected at 2, 6, 10, 14, 18, and 22 h (every 4 h). The x-axis indicates hours after DD onset.)
